# PANCREATODUODENECTOMY AS TREATMENT FOR RECURRENT ACUTE PANCREATITIS DUE TO *PANCREAS DIVISUM*

**DOI:** 10.1590/0102-6720202400040e1834

**Published:** 2024-12-02

**Authors:** Elizeu Bruno Santos SILVA, Maiza Conceição da SILVA, Maria Clara Santos ARAÚJO, Beatriz Melo Santos Lima PAULINO, José Maria Assunção MORAES-JUNIOR, Orlando Jorge Martins TORRES

**Affiliations:** 1 From Universidade Federal do Maranhão, Hospital Universitário Presidente Dutra, Department of Gastrointestinal Surgery, Hepatopancreatobiliary and Liver Transplant Unit - São Luis (MA), Brazil

**Keywords:** Pancreaticoduodenectomy, Pancreas Divisum, Pancreatitis, Cholangiopancreatography, Endoscopic Retrograde., Pancreaticoduodenectomia, Pâncreas Divisum, Pancreatite, Colangiopancreatografia Retrógrada Endoscópica.

## Abstract

**BACKGROUND:**

*Pancreas divisum* is an anatomical abnormality where the junction of the main and accessory pancreatic duct fails to occur and the smaller-caliber duct acts as dominant, resulting in overload during the drainage of the organ’s secretion through the minor duodenal papilla.

**AIMS:**

To report a case of recurrent acute pancreatitis due to symptomatic *pancreas divisum* who underwent pancreatoduodenectomy.

**CASE REPORT:**

A 21-year-old male patient presented with intermittent painful crises, located in the upper abdomen, with radiation to the back, associated with nausea and vomiting, for the past three years. Magnetic resonance imaging and endoscopic retrograde cholangiopancreatography revealed *pancreas divisum*, subsequently confirmed by endoscopic ultrasound. An attempt was made through endoscopic intervention but failed to catheterize the minor papilla; therefore, a pancreaticoduodenectomy was indicated. The organ was identified as hard and atrophied, with moderate peripancreatic inflammation. The histopathological findings also identified a focal well-differentiated G1-type neuroendocrine tumor measuring 0.4 cm.

**CONCLUSIONS:**

In patients with *pancreas divisum*, rare cases may progress to recurrent acute pancreatitis. Pancreaticoduodenectomy is an option in symptomatic patients who had no success with endoscopic treatment.

## RESUMO

**RACIONAL:**

*Pâncreas divisum* é uma anormalidade anatômica em que a junção do ducto pancreático principal e acessório não ocorre, e o ducto de menor calibre atua como dominante, resultando em sobrecarga durante a drenagem da secreção do órgão através da papila duodenal menor.

**OBJETIVOS:**

Relatar um paciente com pancreatite aguda recorrente devido a *pâncreas divisum* sintomático submetido a duodenopancreatectomia.

**RELATO DE CASO:**

Paciente masculino, 21 anos, apresentou crises dolorosas intermitentes, localizadas no abdome superior, com irradiação para o dorso, associadas a náuseas e vômitos, nos últimos três anos. A ressonância magnética e acolangiopancreatografia endoscópica retrógrada revelaram pâncreas divisum, posteriormente confirmado por ultrassonografia endoscópica. Foi feita uma tentativa de intervenção endoscópica, mas não houve sucesso na cateterização da papila menor, sendo indicada duodenopancreatectomia. O órgão foi identificado como duro e atrofiado, com inflamação peripancreática moderada. O resultado histopatológico final também identificou um tumor neuroendócrino focal bem diferenciado do tipo G1, medindo 0,4 cm.

**CONCLUSÕES:**

Em pacientes com pâncreas divisum, casos raros podem progredir para pancreatite aguda recorrente. A pancreatoduodenectomia é uma opção em pacientes sintomáticos que não tiveram sucesso com o tratamento endoscópico.

## INTRODUCTION

*Pancreas divisum* is an anatomical abnormality where the junction of the main and accessory pancreatic duct fails to occur and the smaller-caliber duct acts as dominant, leading to overload during the drainage of the organ’s secretion through the minor duodenal papilla^9^. This anomaly is the most common congenital anatomical variation in the pancreas and occurs in almost 10% of the general population resulting in two separate ducts^4,9,12^. That is an etiology for idiopathic recurrent acute pancreatitis (I-RAP) and chronic pancreatitis. Most patients with such variability are asymptomatic; however, a small percentage may present clinical manifestations of pancreatitis, such as recurrent abdominal pain^3^.

Therapeutic interventions, such as minor papillotomy, stent placement in the dorsal pancreatic duct, or surgical procedures, can significantly benefit patients with symptomatic *pancreas divisum* by alleviating pressure in the main pancreatic duct^11,13^. In a London-based study, it was found that 26% of patients with unexplained pancreatitis, the subgroup classified as idiopathic recurrent pancreatitis, exhibited *pancreas divisum*, in contrast to a prevalence rate of 5.8% observed in the entire group undergoing endoscopic retrograde cholangiopancreatography (ERCP)^6^.

This study reports a case of acute recurrent pancreatitis due to *pancreas divisum* undergoing pancreatoduodenectomy.

## CASE REPORT

**Figure 1:**
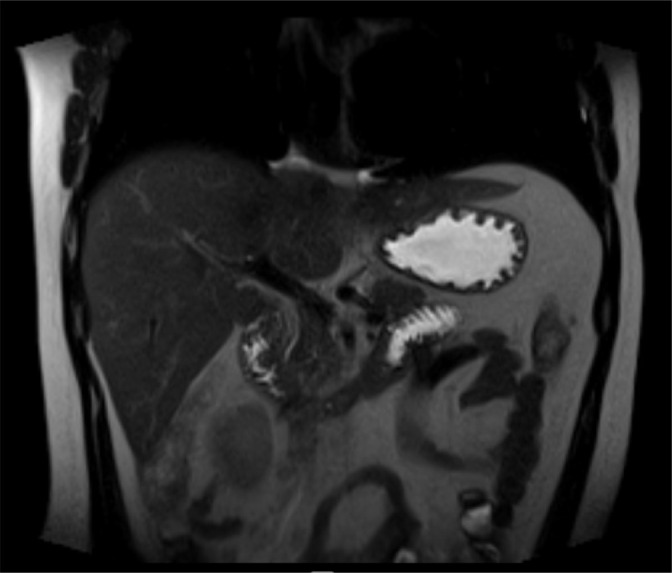
Magnetic resonance image suggesting *pancreas divisum.*

**Figure 2:**
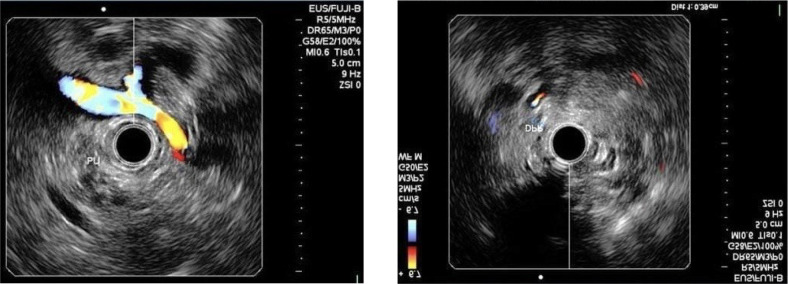
Endoscopic ultrasound confirming *pancreas divisum.*

A 21-year-old male patient presented to the University Hospital President Dutra (HUPD) with intermittent painful crisis for the past three years, localized in the upper abdomen, of strong intensity, abrupt onset, with radiation to the back, associated with nausea, vomiting, constipation, and improvement with the use of analgesics. He had no comorbidities, history of alcoholism, smoking, or allergies. Family history included systemic arterial hypertension, diabetes mellitus, cardiopathy, and an episode of acute pancreatitis due to hypertriglyceridemia. The patient experienced an episode of choluria and denied jaundice and acholic stools four years before. The patient reported multiple visits to the emergency department, one of which included magnetic resonance imaging and cholangiopancreatography (Figure 1), revealing *pancreas divisum*, which was subsequently confirmed by endoscopic ultrasound (Figure 2).

He was referred for outpatient care at HUPD and a physical examination revealed no relevant findings; the cardiovascular and respiratory systems were normal. Upon admission, amylase and lipase levels were within normal limits, but previous evaluations had shown elevation. After one year and multiple episodes of acute pancreatitis, an attempt at endoscopic intervention was made but failed to catheterize the minor papilla. Subsequently, he was admitted with the indication of pancreatoduodenectomy.

**Figure 3:**
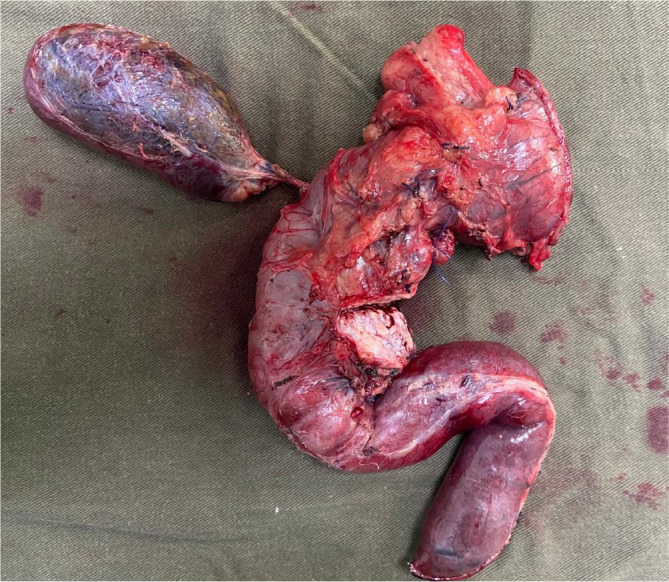
Surgical specimen of pancreatoduodenectomy.

After preoperative evaluation, the pancreatoduodenectomy was performed and the pancreas was identified as hard and atrophied, with moderate peripancreatic inflammation. The specimen was removed (Figure 3) and anastomosis was performed using the Heidelberg technique modified by Torres et al.^12^.

The patient stayed in the intensive care unit (ICU) for three days and was sent home on the seventh postoperative day. At the outpatient follow-up, he reported an episode of non-bloody diarrhea, but there was spontaneous recovery, and the postoperative course was uneventful. The histopathological findings confirmed the presence of a focal well-differentiated G1-type neuroendocrine tumor measuring 0.4 cm. Additionally, it was observed that ductal drainage occurred separately into the major and minor duodenal papillae, consistent with the *pancreas divisum.* The patient signed a consent form authorizing this report.

## DISCUSSION

*Pancreas divisum* is an anatomical anomaly characterized by separate pancreatic ducts, with the secondary Santorini duct taking precedence over the Wirsung duct, as seen in normal anatomical development. This anomaly results in stagnant organ secretion, leading to continuous injury to the parenchyma and inflammation^8^. Acute recurrent pancreatitis develops in 5% of patients with the condition^1^. Understanding this possibility is not recent and has already been reported. The reduced diameter of the Santorini duct does not allow proper drainage of pancreatic secretion and causes tissue injury^10,14^.

The symptomatology associated with *pancreas divisum* typically manifests as acute pain in the epigastric region^5^. As the condition progresses, the frequency and intensity of this pain contribute to the chronicity of the disease, leading to the gradual destruction of pancreatic acini, which can be observed through changes in imaging exams. While laboratory parameters often demonstrate elevated levels of pancreatic enzymes during acute episodes, their reduction does not necessarily indicate the absence of pancreatitis but rather suggests organ failure^7,8^.

Endoscopic ultrasound contributes to adequate visualization and allows for the early detection of pancreatitis^14^. Through this modality, clinicians can identify hallmark signs such as atrophy, heterogeneity of the entire parenchyma, and diffuse hyperechoic streaks. These findings are under criteria established previously and classification for detecting the alteration was studied through this examination^5^.

The surgical specimen revealed a hard and atrophic pancreas, as continuous destruction of parenchymal cells leads to replacement by fibrotic tissue^11^. Microscopic analysis identified focal cellular proliferation consistent with neoplasia. The identified tumor affects the pancreas and is defined as a “pancreatic neuroendocrine tumor” when limited to it^11^. Neoplastic tissue can release hormones and cause symptoms related to the action of the secreted molecule, or not. In this case, no symptoms were manifested, and the patient presented a well-differentiated neuroendocrine tumor, G1-type, a classification described in the literature as a lower grade of malignancy^1,3,14^. Some previous reports described the association between early surgery as the first therapeutic choice and more satisfactory future outcomes. However, the initially proposed treatment is ERCP, as this procedure is less invasive and usually better accepted by patients^11^. If the endoscopic procedure fails, surgical therapy is indicated^2,12^ such as pancreatoduodenectomy^2^, depending on the clinical case and anatomical variation. In the present study, the choice for pancreatoduodenectomy occurred after ERCP failure.

## CONCLUSIONS

In patients with *pancreas divisum*, rare cases are symptomatic and may progress to recurrent acute pancreatitis. Pancreatoduodenectomy is an option in those patients who had no success with endoscopic treatment.
